# Burden and well-being among dementia caregivers in Puerto Rico: the role of behavioral and psychological symptoms of dementia

**DOI:** 10.1093/geronb/gbag014

**Published:** 2026-02-05

**Authors:** Junyub Lim, Ross Andel, Frank Puga, María P Aranda, Maricruz Rivera-Hernandez, Ana Luisa Dávila-Roman, Michael Crowe

**Affiliations:** Edson College of Nursing and Health Innovation, Arizona State University, Phoenix, Arizona, United States; Edson College of Nursing and Health Innovation, Arizona State University, Phoenix, Arizona, United States; School of Nursing, University of Alabama at Birmingham, Birmingham, Alabama, United States; Suzanne Dworak-Peck School of Social Work, Edward R. Roybal Institute on Aging, University of Southern California, Los Angeles, California, United States; Department of Health Services, Policy, and Practice, Brown University School of Public Health, Providence, Rhode Island, United States; School of Public Health, University of Puerto Rico, San Juan, Puerto Rico; Department of Psychology, University of Alabama at Birmingham, Birmingham, Alabama, United States; (Psychological Sciences Section)

**Keywords:** Depressive symptoms, Social support, Hispanic/Latino, Caregiver burden, Appetite/eating difficulties in people living with dementia

## Abstract

**Objectives:**

Dementia is more prevalent in Puerto Rico than in the U.S. mainland, increasing demands for caregiving. However, research on the mental health impact of dementia caregiving among island Puerto Ricans is sparse. We examined caregiver burden and depressive symptoms among Puerto Rican dementia caregivers while also considering behavioral and psychological symptoms of dementia (BPSD).

**Methods:**

Using data from the Puerto Rican Elder: Health Conditions (PREHCO) study (Wave 3), 132 caregivers of PREHCO participants requiring care completed an ancillary data collection, of whom 101 cared for PREHCO participants with dementia. Measures included the 15-item Geriatric Depression Scale (depressive symptoms), Neuropsychiatric Inventory Questionnaire (NPI-Q; BPSD), and Zarit Burden Interview (ZBI-6; caregiver burden).

**Results:**

Caregivers were 63 ± 10 years of age on average, and 77% were women. Exploratory factor analysis yielded two BPSD factors: Emotional Dysfunction/Behavioral Instability and Perceptual Disturbances/Apathy. In linear regression models adjusted for age, sex, education, marital status, self-rated health, hours of caregiving per week, and perceived social support, caregiver burden was positively associated with depressive symptoms (*β* = 0.68, *p* < .001), as were BPSD factors Emotional Dysfunction/Behavioral Instability (*β* = 0.50, *p* < .001) and Perceptual Disturbances/Apathy (*β* = 0.50, *p* < .001). Only Perceptual Disturbances/Apathy significantly moderated this association (*β* = 0.24, *p* = .032), with the burden–depression link strengthening as BPSD increased. Appetite/Eating Symptoms (*β* = 0.42, *p* = .007) emerged as the main BPSD item driving this moderation effect.

**Discussion:**

In this Puerto Rico-based sample, perceptual disturbances/apathy, and appetite/eating symptoms specifically, were most likely to magnify the caregiver burden-depressive symptoms link.

Caregiving for people living with dementia is among the most demanding unpaid tasks. While it can be rewarding, it may also have serious negative consequences for mental health and quality of life for caregivers (Ravyts & Dzierzewski, 2024). Among known caregivers, 28% report caring for individuals with dementia (Centers for Disease Control and Prevention, 2021). Previous research suggests that dementia caregivers exhibit high levels of depressive symptoms (Haley et al., 1987; Roth et al., 2008), and about a third have clinical depression (Huang, 2022). Dementia represents the fourth most common cause of death in Puerto Rico (Friedman et al., 2016), with dementia prevalence exceeding 10% among older adults over 65 years old (NORC at the University of Chicago, 2024). Still, little is known about crucial factors influencing mental health outcomes among Puerto Rican dementia caregivers. The socioeconomic conditions in Puerto Rico, where high levels of outmigration have reduced the pool of available kin who might otherwise share caregiving responsibilities, and where older adults face more limited and lower-quality healthcare despite high insurance coverage, underscore the importance of studying dementia care within this population (Matos-Moreno et al., 2022). We set out to address this gap by examining both caregiver and care recipient characteristics in relation to the mental health of dementia caregivers residing in Puerto Rico.

Dementia caregivers are at a higher risk of experiencing depressive symptoms due to care burden compared to non-dementia caregivers (Pinquart & Sörensen, 2003), likely stemming from a combination of limited personal time, increased likelihood of family conflict, employment complications, worsened physical health, and lower overall quality of life (Pinquart & Sörensen, 2003). On the other hand, caregiving may offer the caregiver a sense of purpose in life, self-satisfaction, and engagement with a loved one that may buffer against poorer mental health outcomes (Marino et al., 2017). Results from a recent study also suggested that caregivers may have better cognitive functioning than non-caregivers when matched for sociodemographic and health characteristics (Elayoubi et al., 2023). Given the evidence that caregiver outcomes may vary widely (Li et al., 2023), it is necessary to identify the specific challenges within dementia care, particularly where family support may be expected, in order to develop more targeted interventions. Thus, we examined whether dementia symptoms affect caregiver burden and subsequently affect depressive symptoms in the caregiver.

## Behavioral and psychological symptoms of dementia

Behavioral and psychological symptoms of dementia (BPSD) encompass a diverse range of neuropsychiatric behaviors and symptoms, such as agitation, anxiety, depression, and apathy, commonly observed in older adults with dementia (Cerejeira et al., 2012). BPSD can increase dementia caregiver burden, which may potentially affect caregiver well-being. In a systematic review, anger/aggression and depression were most frequently identified symptoms associated with caregiver burden (Ornstein & Gaugler, 2012). According to Feast et al. (2016), communication changes caused by BPSD in people living with dementia also contribute substantially to caregiver burden, making previously enjoyable conversations with family caregivers difficult or, in severe cases, leading to a complete lack of interaction. However, it remains unclear whether BPSD heightens negative caregiving appraisal and, in turn, strengthens the association between caregiver burden and adverse mental health outcomes.

It is important to note that while BPSD include a wide spectrum of behaviors and symptoms, these tend to co-occur and mutually reinforce one another. For this reason, BPSD have been examined at the group rather than the individual symptom level using data reduction techniques (Van Der Linde et al., 2014). For example, using factor analysis, Kim et al. (2021) found factors related to hyperactivity symptoms, psychosis symptoms, and physical behavior symptoms associated with BPSD, with each of these factors showing a significant direct effect on caregiver burden.

Although previous studies have consistently demonstrated a significant positive association between BPSD and caregiver burden (Pinquart & Sörensen, 2003), there is limited understanding of the interaction between caregiver burden and BPSD in relation to caregivers’ mental health. A more detailed understanding of how BPSD interact with caregiver burden can help identify more vulnerable subgroups, which in turn can help both manage caregiving expectations and provide targeted support where it is likely most needed. BPSD symptoms may be especially burdensome for Puerto Rican dementia caregivers, who often navigate these challenges in cultural contexts that prioritize family obligation over formal care (Yeo & Gallagher-Thompson, 2014). This may lead to greater reliance on family support and reduced use of professional mental health services, even when specialized care is needed.

## The sociocultural stress and coping model

The interaction between BPSD and caregiver burden can be conceptualized using the Sociocultural Stress and Coping Model (SSC model, Knight & Sayegh, 2010). The model explains how challenges in caring for individuals with dementia, mediated by perception of a high caregiver burden, are negatively associated with caregivers’ physical and mental health. The SCC model is based on Lazarus and Folkman’s (1984) conceptual framework, which helps outline the interrelationships among stressors encountered in caregiving (e.g., the BPSD of people living with dementia), caregivers’ appraisal of these stressors (e.g., caregiver burden), coping responses, social support, and caregiver mental health outcomes. Knight and Sayegh (2010) expanded this model by suggesting that culturally specific values (e.g., filial piety, familism) influence social support and coping styles, which can alter how caregiver burden from the care recipient’s condition affects caregivers’ health outcomes.

The SSC model’s emphasis on culturally specific values is evident in findings that African American caregivers, who exhibit stronger familism compared to white caregivers, reported greater social support, which indirectly contributed to lower levels of caregiver burden and depression (Falzarano et al., 2022). Similarly, in a study of Hispanic and non-Hispanic White caregivers from the San Francisco, CA, area, Hispanic dementia caregivers demonstrated higher self-efficacy in managing upsetting thoughts related to caregiving compared to non-Hispanic White caregivers, which served as a significant mediator of lower caregiver burden in the Hispanic caregivers (Montoro-Rodriguez & Gallagher-Thompson, 2009). These findings go along well with the SSC model, as presumably, caregivers from tighter-knit Hispanic families may perceive less dementia caregiver burden than White dementia caregivers.

## Hispanic culture and dementia caregiving

Hispanic dementia caregivers encounter both unique advantages and challenges that are at least partially shaped by sociocultural norms. Two of the most frequently cited cultural values in Hispanic caregiving research are *familism*—which depicts strong identification with and loyalty to family—and marianismo—which emphasizes women’s self-sacrificing devotion to familial responsibilities (Arévalo-Flechas et al., 2014). Familism specifically has been described as a cultural resource that may buffer caregiving stress among Hispanic caregivers (Mausbach et al., 2004). However, the influence of familism is not uniform across Hispanic dementia caregivers. For some, familism creates a sense of obligation to take on a caregiving role regardless of personal capacity (Gelman, 2014). In Puerto Rico specifically, this notion can be exacerbated by dissipating social networks due to migration and economic pressures (Matos-Moreno et al., 2022). When familism intersects with gender role expectations, such as marianismo, the physical and emotional burden on female caregivers may be further intensified (Arévalo-Flechas et al., 2014). In such contexts—where Hispanic cultural values may reinforce rather than alleviate caregiver burden—the presence of accessible social support can be critical (Aranda, 2001). This sociocultural dynamic becomes particularly relevant in the Puerto Rican context, where policy and economic challenges (Matos-Moreno et al., 2022) and cultural norms (Yeo & Gallagher-Thompson, 2014) can lead older adults to rely more heavily on informal care provided by family members. In this context, the need for social support for primary caregivers becomes even more critical. Caregivers often share caregiving responsibilities with siblings or seek both emotional and practical support from community resources, such as churches (Aranda, 2001). Despite the recognized importance of social support for Puerto Rican dementia caregivers, there is limited research that has quantitatively examined its relationship with dementia-related caregiver burden and caregivers’ mental health.

## Current study

Building on previous research, we aimed to examine the relationship between caregiver burden and depressive symptoms among dementia caregivers in Puerto Rico, a unique setting shaped by distinct sociocultural caregiving norms. We also assessed whether BPSD factors, or any individual BPSD, would significantly modify any initially observed association between caregiver burden and depressive symptoms (Supplementary Figure 1). Finally, we sought to explore whether perceived social support would partially explain the association between caregiver burden and depressive symptoms. We hypothesized that:

1. Greater caregiver burden would be associated with higher depressive symptoms.
2. Higher levels of BPSD would strengthen the positive association between caregiver burden and depressive symptoms.
3. Perceived social support would reduce the magnitude of the relationship between caregiver burden and depressive symptoms.

## Method

### Participants

**Figure 1:**
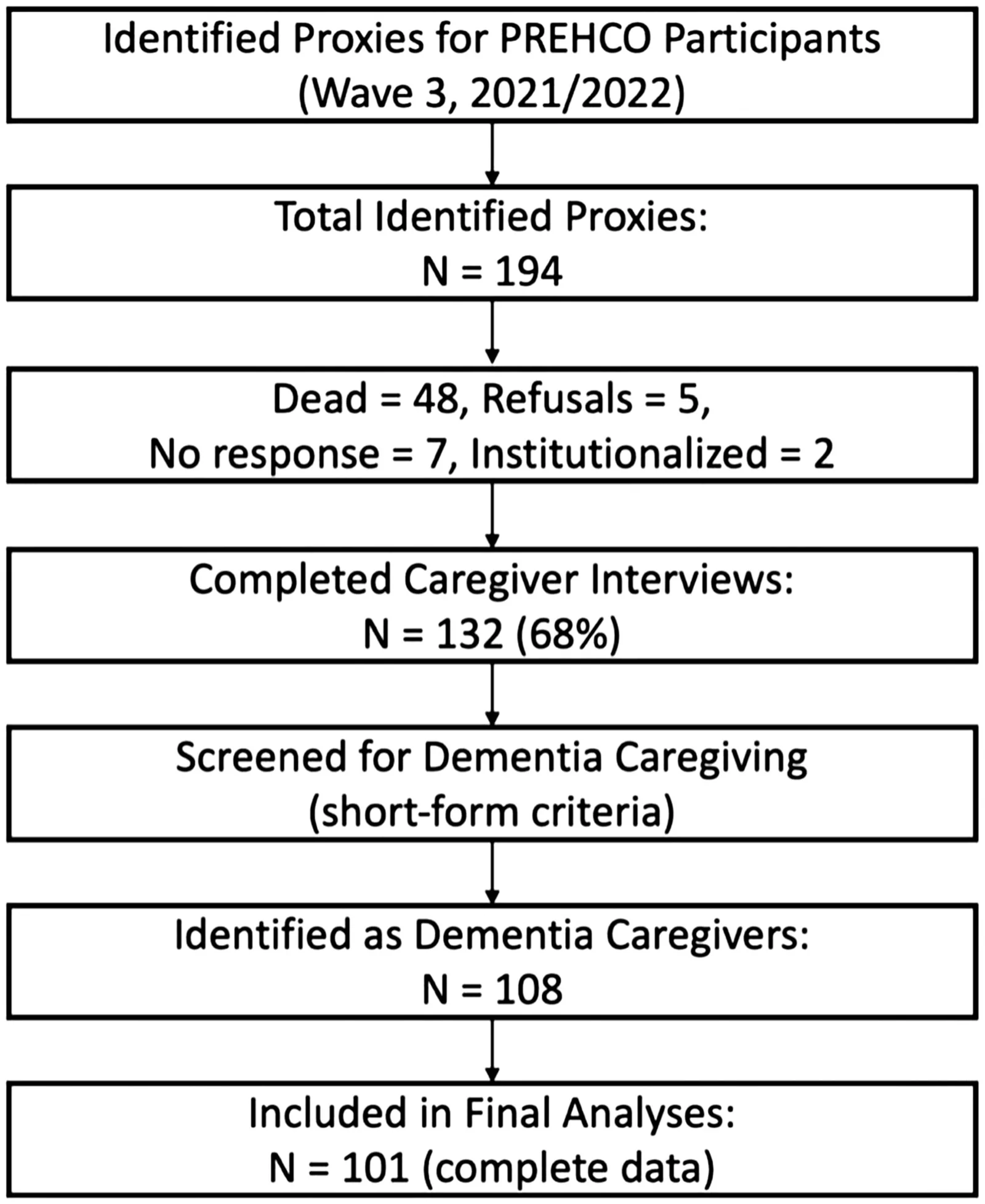
Participant flow diagram for the PREHCO dementia caregiver study.

Participants were informant proxies for members of the Puerto Rican Elder: Health Conditions (PREHCO) Study (Palloni et al., 2005) who were part of the 2021/2022 data collection (Wave 3; see Figure 1 for the participant flowchart). The PREHCO study is a representative longitudinal survey of older adults in Puerto Rico, which originated in 2002. In Wave 3, PREHCO participants were aged 78 years and older, and an ancillary PREHCO data collection was developed for caregivers of PREHCO participants who also served as proxies for the main study. Initially, 194 proxies were identified, of whom 132 were a caregiver for the participant and completed the ancillary interview (68%).

Since this study focused specifically on caregivers of people living with dementia, the 132 caregivers who completed the interviews were further classified as dementia caregivers or non-dementia caregivers based on informant reports. To identify caregivers of PREHCO participants with dementia, we used the Health and Retirement Study (Crimmins et al., 2011) version of the Informant Questionnaire on Cognitive Decline in the Elderly (IQCODE; Jorm, 1994), which asked caregivers about changes over the past two years in memory and everyday function of the older adults for whom they cared. Based on the criteria established by Mejía-Arango et al. (2020), a score of 3.4 or higher indicated that the older adult was likely experiencing dementia, thereby classifying the caregiver as a dementia caregiver. In all, 108 caregivers were identified as caring for people living with dementia. Of these, 101 caregivers with complete information on all key variables used in the current study were included in final analyses. The internal review boards at University of Puerto Rico and University of Alabama at Birmingham approved the original data collection. The internal review board at Arizona State University approved the analyses of de-identified data conducted by the study authors.

### Measures

#### Dependent variable

Depressive symptoms were measured using the Geriatric Depression Scale (GDS), the 15-item version, with binary responses (yes/no) (Sheikh & Yesavage, 1986). To ensure that higher scores indicate greater depressive symptomatology, four of the 15 items were reverse-coded, and possible total scores range from 0 to 15. The GDS has been widely used in previous studies to assess depressive symptoms in dementia caregivers (Gaugler et al., 2018), higher scores reflect higher depressive symptoms. In this study, the 15-item GDS demonstrated acceptable internal consistency (Cronbach’s *α* = 0.74), and its psychometric properties have been validated in U.S. Latino informal caregivers (Perales-Puchalt et al., 2025).

#### Independent variable

Caregiver burden was measured using the 6-item Zarit Burden Interview (ZBI). Originally introduced as a 29-item scale (Zarit et al., 1985), the ZBI was later revised to a 22-item version, which became widely used. To facilitate quicker administration, various shorter versions (e.g., ZBI-12, ZBI-8, ZBI-7, ZBI-6, and ZBI-4) were developed. Among these, the ZBI-6, used in this study, has been shown to have the minimal number of items while maintaining diagnostic utility close to the 22-item version (Yu et al., 2019). Each item was measured on a five-point scale from 0 (never) to 4 (very often). Caregiver burden was calculated as the total score across six items, ranging from 0 to 24, with higher scores indicating greater burden. The scale demonstrated good internal consistency in the present study (Cronbach’s *α* = 0.84). The ZBI-6 has likewise been validated with good psychometric properties among U.S. Latino informal caregivers (Perales-Puchalt et al., 2025).

#### Moderator variable

Behavioral and Psychological Symptoms of Dementia were measured using the Neuropsychiatric Inventory Questionnaire (NPI-Q) (Kaufer et al., 2000). The NPI-Q assesses BPSD across 12 symptom areas—delusions, hallucinations, depression, anxiety, apathy, irritability, elation, disinhibition, agitation, aberrant motor behavior, appetite and eating disturbance, and nighttime behavioral disturbances, with responses collected based on caregivers’ reports. Caregivers responded to each domain question with “yes” or “no” to indicate the presence or absence of symptoms. If symptoms are present, their severity is rated as 1 = mild, 2 = moderate, or 3 = ­severe. In this study, each domain was coded as a single variable for factor analysis of BPSD. A response of “no” was coded as 0, while a response of “yes” was coded based on severity, ranging from 1 (mild) to 3 (severe). Therefore, each of the 12 domains has a possible score range of 0–3.

##### Other variables

Based on prior research indicating their influence on caregiver appraisals and mental health, participants’ demographic and health-related characteristics and an indicator of caregiving demand were included as covariates (Perkins et al., 2013), including age (years), sex (0 = male, 1 = female), educational attainment (0 = less than high school, 1 = high school, 2 = associate/technical degree, 3 = bachelor’s degree or higher), marital status (0 = without a partner, 1 = married/partnered), self-rated health (0 = poor/fair, 1 = good, 2 = very good/excellent), and weekly caregiving hours (0 = 35 hr or less, 1 = more than 35 hr up to 100 hr, 2 = more than 100 hr). Perceived social support was included to test whether this variable may partially explain the association between caregiver burden and depressive symptoms. It was assessed using the Lubben Social Network Scale (LSNS-6; Lubben et al., 2006) which measures caregivers’ perceived support from family and friends. Three questions were asked separately for each group: (1) How many people do you see or hear from at least once a month? (2) How many people do you feel at ease with to discuss private matters? (3) How many people do you feel close to and could call on for help? Responses to each question were recorded using six options: 0 (none), 1 (one), 2 (two), 3 (three or four), 4 (five to eight), and 5 (nine or more). Higher total scores indicate greater levels of perceived social support, with a range of 0–30, and the scale demonstrated acceptable internal consistency (Cronbach’s *α* = 0.77).

### Analyses

Descriptive statistics for the participants were used to summarize the characteristics of variables. The 12 BPSD domains measured by the NPI-Q were examined using exploratory factor analysis (EFA) followed by confirmatory factor analysis (CFA). To enhance estimation accuracy, given that the BPSD were measured as ordinal variables, a polychoric correlation matrix was used for both factor analyses. Before conducting EFA, suitability of data was assessed using the Kaiser-Meyer-Olkin (KMO) test and Bartlett’s test of sphericity, with a KMO value of 0.60 or higher and a significant Bartlett’s test (*p* < .05) indicating suitability for factor analysis. The number of factors was determined using the criterion of eigenvalues greater than 1.0 and by examining the scree plot for a sharp drop in eigenvalues between factors. Items were subsequently assigned to factors using a minimum loading threshold of 0.40. CFA was performed by linking each BPSD item derived from EFA, employing the weighted least square mean and variance-adjusted (WLSMV) estimator, which is appropriate for polychoric correlations (Finney & DiStefano, 2006). Model fit was assessed using comparative fit indices (CFI), Tucker–Lewis index (TLI), and RMSEA, with CFI and TLI values greater than 0.95 and an RMSEA value less than 0.06 indicating good model fit (Hu & Bentler, 1999). Since CFA was conducted using a polychoric correlation matrix, scaled goodness-of-fit indices were used to evaluate model. Finally, the factor scores obtained from CFA were standardized and used in the main analyses.

Multivariable ordinary least square (OLS) regression analyses were conducted to test the research hypotheses. Age, sex, educational attainment, marital status, self-rated health, weekly caregiving hours, and perceived social support were included as covariates sequentially. In Model 1, age, sex, education, marital status, and self-rated health were controlled for. In Model 2, hours of caregiving per week were additionally adjusted. Finally, we added perceived social support in Model 3 to test whether it may explain away any association between caregiver burden and depressive symptoms as per our third hypothesis.

Finally, we examined the interaction effects between each BPSD-based factor and caregiver burden separately. Standardized coefficients were reported to increase interpretability and allow comparisons of the magnitude of associations across models. The Johnson-Neyman technique (Johnson & Fay, 1950) was used to determine the specific range of observed BPSD factor scores within which the association between caregiver burden and depressive symptoms was statistically significant.

All data preprocessing and statistical analyses were performed using R version 4.4.1. Moderation analyses were conducted using the *interactions* package (version 1.2.0). The first author used ChatGPT to review the completed manuscript for English translation and readability. The content was subsequently reviewed and further edited by all authors.

## Results

The sociodemographic characteristics of the caregivers are presented in Table 1. The dementia caregivers had a mean age of 63 years (*SD* = 11 years), and the majority were female (77%). Most participants had completed at least a high school education (90%), and slightly more than half had a spouse or partner. About half of the participants reported their health as poor or fair. Most caregivers were the adult children of the care recipient (75%) and reported providing an average of around 100 hr of care per week. Table 2 presents the frequency of BPSD along with the severity distribution among those who reported each behavior or symptom. Participants reported that care recipients exhibited an average of five concurrent BPSD symptoms, with nighttime behavior being the most frequently reported symptom (*n* = 65) and delusions being the least frequent (*n* = 22).

**Table 1:** Characteristics of caregivers in the study sample (*N* = 101).

Variable	Mean	*SD*	Range	%
**Age**	63.0	10.9	28–87	
**Female**				77.2
**Educational attainment**				
** Less than high school**				9.9
** High school**				43.6
** Associate degree or technical degree**				18.8
** Bachelor’s degree or higher**				27.7
**Married or partnered**				54.5
**Self-rated health**				
** Poor or fair**				54.5
** Good**				32.7
** Very good or excellent**				12.9
**Relationship with care-recipient**				
** Spouse or partner**				9.9
** Son or daughter**				75.2
** Others**				14.9
**Weekly caregiving hours**	99.4	60.1	5–168	
** Less than 35**				19.8
** Between 35 and 100**				29.7
** More than 100**				50.5
**Number of concurrent BPSD**	4.9	3.1	0–12	
**Perceived social support**	14.0	5.8	3–30	
**Caregiver burden**	7.9	6.3	0–24	
**Depressive symptoms**	4.3	2.8	1–12	

*Note. SD* = standard deviation; BPSD = behavioral and psychological symptoms of dementia.

**Table 2:** Frequency of BPSD by severity levels.

Symptoms	Yes	Mild	Moderate	Severe
**Nighttime behavior**	65	8	37	20
**Apathy/indifference**	54	16	20	18
**Agitation/aggression**	53	17	19	17
**Appetite/eating**	46	14	17	15
**Irritability/lability**	43	14	16	13
**Dysphoria/depression**	43	22	15	6
**Aberrant motor**	39	10	20	9
**Hallucinations**	38	12	15	11
**Disinhibition**	37	18	8	11
**Anxiety**	31	7	9	15
**Euphoria/elation**	26	11	13	2
**Delusions**	22	7	9	6

*Note.* BPSD = behavioral and psychological symptoms of dementia.

Supplementary Table 1 shows the polychoric correlation ­matrices derived from the 12 NPI-Q items. Exploratory factor analysis identified two BPSD factors, which were generally represented by emotional dysfunction/behavioral instability (eigenvalue = 3.51, variance = 0.29) and perceptual disturbances/apathy (eigenvalue = 2.03, variance = 0.17). Using a minimum loading threshold of 0.4, 7 of the 12 BPSD items loaded onto emotional dysfunction/behavioral instability in the following order of ­loading magnitude: delusions, disinhibition, agitation/aggression, ­irritability/lability, aberrant motor behavior, anxiety, and nighttime behavior. Four BPSD items loaded onto perceptual disturbances/apathy, in the following order: dysphoria/depression, appetite/eating disturbances, apathy/indifference, and hallucinations. Cronbach’s *α* was 0.80 for emotional dysfunction/behavioral instability and 0.71 for perceptual disturbances/apathy. Among the 12 BPSD items, euphoria/elation had loadings below 0.4 on both factors (Supplementary Table 2). Since it was excluded from the CFA due to low factor loadings, the symptom was also not included in the OLS analyses. CFA of the two-factor model derived from EFA yielded *χ*^2^(43) = 56.44, CFI = 0.97, TLI = 0.96, and RMSEA = 0.06 (90% CI: 0.00, 0.09), supporting the model’s adequacy. The correlations among all study variables, including the BPSD factors derived from factor analysis, are summarized in Supplementary Table 3.

Table 3 presents the main associations between caregiver burden and the two BPSD factors and the outcome of depressive symptoms. The results indicated that higher caregiver burden and each of the BPSD factors were significantly and positively associated with depressive symptoms across all three models. In addition, the estimates for caregiver burden and the two factors in relation to depressive symptoms remained consistent after the inclusion of weekly caregiving hours in Model 2 and the inclusion of perceived social support in Model 3, suggesting that perceived social support had no significant mitigating effect on the magnitude of these associations.

**Table 3:** Main effects and interaction effects for caregiver burden and BPSD in relation to caregiver depressive symptoms (*N* = 101).

Variable	Model 1				Model 2				Model 3			
	Est.	*SE*	*β*	*p*	Est.	*SE*	*β*	*p*	Est.	*SE*	*β*	*p*
**Intercept**	−1.20	1.26	0.00	.344	−1.09	1.28	0.00	.397	−0.21	1.34	0.00	.873
**Caregiver burden**	0.31	0.03	0.70	<.001	0.32	0.03	0.70	<.001	0.30	0.03	0.68	<.001
**BPSD Factor 1**	1.45	0.25	0.51	<.001	1.46	0.25	0.51	<.001	1.41	0.24	0.50	<.001
**BPSD Factor 2**	1.47	0.24	0.52	<.001	1.49	0.25	0.53	<.001	1.42	0.24	0.50	<.001
**Caregiver burden × Factor 1**	0.05	0.03	0.18	.104	0.05	0.03	0.19	.095	0.04	0.03	0.15	.169
**Caregiver burden × Factor 2**	0.06	0.03	0.23	.035	0.06	0.03	0.24	.032	0.05	0.03	0.21	.060

*Note.* BPSD = behavioral and psychological symptoms of dementia; Est. = estimate (unstandardized regression coefficient); *SE* = standard error; *β* = standardized regression coefficient; Factor 1 = emotional dysfunction/behavioral instability; Factor 2 = perceptual disturbances/apathy. Caregiver burden and the two factors were included in separate models. Each factor score was standardized using *z*-scores. The intercept values are for the model with caregiver burden as the independent variable. Model 1 was adjusted for age, sex, education, marital status, and self-rated health; Model 2 was also adjusted for hours of caregiving per week; Model 3 was also adjusted for perceived social support.

**Figure 2:**
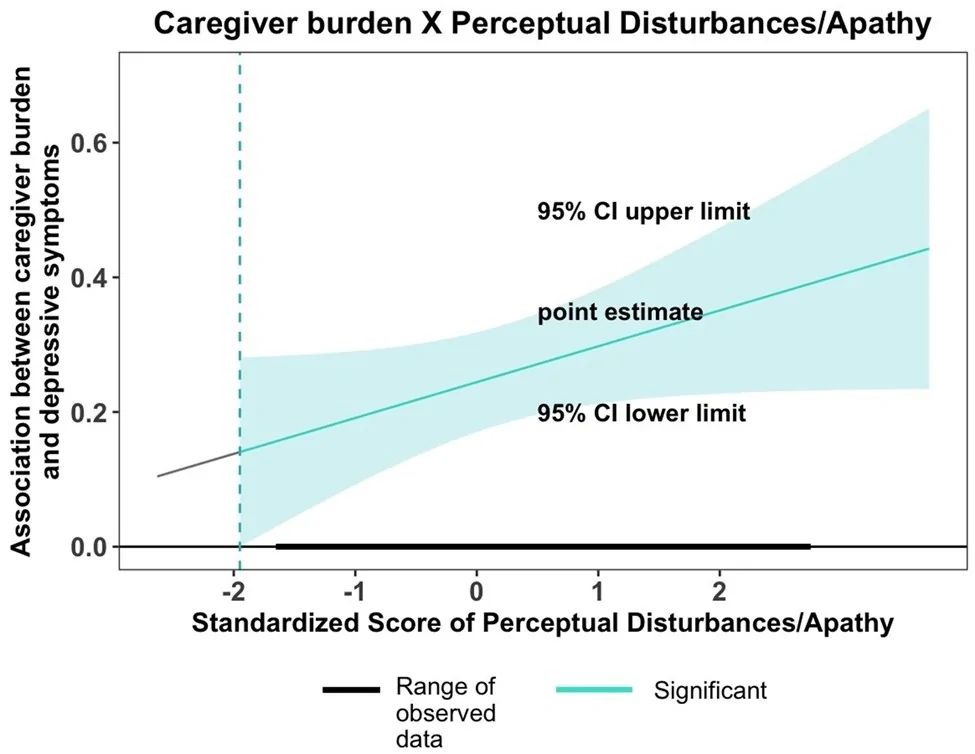
Association between caregiver burden and depressive symptoms, with perceptual disturbances/apathy as a moderator. *Note*. The region of significance from the Johnson-Neyman analysis is highlighted; The graph above corresponds to Model 3 and presents results adjusted for covariates including age, sex, education, marital status, self-rated health, hours of caregiving per week, and perceived social support.

Finally, we examined the moderating effects of each BPSD factor on the association between caregiver burden and depressive symptoms (see also Table 3). Only perceptual disturbances/apathy showed a significant moderating effect in Models 1 and 2 (*β* = 0.23, *p* < .05; *β* = 0.24, *p* < .05), and a non-significant effect in Model 3 (*β* = 0.21, *p* = .060), such that the association between caregiver burden and depressive symptoms became stronger as BPSD symptoms reflected in perceptual disturbances/apathy increased. The association between caregiver burden and depressive symptoms was statistically significant across the entire spectrum of perceptual disturbances/apathy scores (see Figure 2).

In post hoc analyses, we sought to obtain a better understanding of potential drivers behind the observed moderation effect by conducting follow-up analyses using the individual BPSD ­symptoms with the highest loadings on the perceptual disturbances/apathy factor: dysphoria/depression (loading = 0.73) and appetite/eating (loading = 0.60; Supplementary Table 2). Only appetite/eating consistently showed a statistically significant moderating effect across all three models (Model 1: *β* = 0.43, *p* < .01; Model 2: *β* = 0.42, *p* < .01; Model 3: *β* = 0.37, *p* < .05), indicating that the association between caregiver burden and depressive symptoms became markedly stronger as appetite/eating problems increased (Supplementary Table 4). The standardized coefficient *β* for the moderating effect of the appetite/eating symptom alone was also nearly twice the size observed for perceptual disturbances/apathy (0.21 vs. 0.37).

## Discussion

The purpose of this study was to examine the association between caregiver burden and depressive symptoms in dementia caregivers living in Puerto Rico, and to examine the role of BPSD and social support in this association. There were three major findings in our study. First, we found that caregiver burden and both BPSD factors were significantly and positively associated with depressive symptoms across all models, even after adjusting for sociodemographic characteristics, caregiving hours, and perceived social support. Second, the BPSD factor represented by perceptual disturbances/apathy significantly moderated (strengthened) the relationship between caregiver burden and depressive symptoms, and the effect appeared to be driven mainly by appetite/eating symptoms. Moderation was not observed for the factor representing emotional dysfunction/behavioral instability. Finally, perceived social support did not influence the results. These findings provide new evidence for the association between caregiver burden and depressive symptoms in Puerto Rico, highlight that BPSD, particularly appetite/eating problems, may lead to poorer mental health among caregivers, and that, in this culturally unique sample of dementia caregivers, social support may not play the expected role in the association between caregiver burden and depressive symptoms.

The finding that caregiver burden and both BPSD factors were significantly and positively associated with depressive symptoms across all models suggests that negative perceptions of the caregiving experience and the symptoms shown by the person with dementia are strong and independent predictors of depressive symptoms in caregivers. Notably, the correlation coefficient between caregiver burden and depressive symptoms (*r* = 0.72) was higher than the correlation (*r* = 0.52) reported in a systematic review (del-Pino-Casado et al., 2019), suggesting a stronger association in this sample.

We found that BPSD reflecting perceptual disturbances/apathy moderated the association between caregiver burden and depressive symptoms, with post hoc analyses indicating that this effect was largely driven by appetite/eating symptoms. These findings suggest that specific symptoms within the perceptual disturbances/apathy, particularly those related to appetite and eating, may play a pivotal role in amplifying the negative appraisal of caregiving. The finding that appetite/eating was the most significant moderator of BPSD is intriguing. We can speculate that this BPSD item may serve as a proxy for more intensive, hands-on caregiving, given the eating assistance needed to address this symptom. Regardless, appetite/eating difficulties in people living with dementia may deserve additional attention in attempts to reduce caregiver burden among Hispanic dementia caregivers.

Emotional dysfunction/behavioral instability did not moderate the association between caregiver burden and depressive symptoms. This suggests that while these symptoms might directly contribute to caregiver distress, they may not necessarily alter the caregiver’s overall negative appraisal of the caregiving experience itself.

In our study, perceived social support did not play a role in the association between caregiver burden and depressive symptoms. This finding contrasts with prior studies reporting buffering effects of perceived support on caregiver burden and depressive symptoms, and therefore warrants careful interpretation (del-Pino-Casado et al., 2018; Gutiérrez-Sánchez et al., 2023). One possible explanation is that the LSNS-6 may not have captured the construct of social support relevant to caregiving contexts, as it assesses the size of one’s social network rather than the actual receipt or use of support. Indeed, for this reason, del-Pino-Casado et al. (2018) excluded the LSNS-6 from their review. In addition, within Hispanic caregiving culture—particularly under familism and marianismo—merely having access to social ties does not necessarily translate into experienced support. Familism may create an expectation of family support that is not always realized in practice (Gelman, 2014), and marianismo may discourage women from requesting or utilizing available help by reinforcing self-sacrificial caregiving norms. Thus, even when social network resources are available, they may not manifest as meaningful support that alleviates caregiving stress. These cultural mechanisms provide a plausible explanation for why perceived support, as operationalized in this study, did not attenuate the association between burden and depressive symptoms.

Importantly, these culturally shaped caregiving norms operate within a broader structural context in Puerto Rico, where traditional family support systems have been weakened. Due to migration to the U.S. mainland, low fertility rates, and increasing life expectancy, as well as economic factors (Matos-Moreno et al., 2022), Puerto Rico is experiencing low levels of social support and high levels of social isolation (Gao et al., 2021). Thus, a combination of the gap between culturally expected and actually available family support, caregiving role norms that place primary responsibility on family members, particularly women, and broader structural conditions that limit the availability of support may jointly explain why perceived social support, as measured by network size rather than enacted support, did not attenuate the relationship between caregiving stress and depressive symptoms in this study.

These findings, considered in the broader sociocultural and demographic context of Puerto Rico, suggest several potential directions for caregiver support. First, appetite and eating difficulties may reflect a set of caregiving demands that are highly hands-on and time-intensive. Interventions that are symptom-focused and provide both skills training and opportunities for task sharing or respite may help reduce the extent to which these symptoms shape caregiving appraisal negatively, thereby mitigating adverse mental health outcomes. Second, although this study could not determine whether actual received support reduces depressive symptoms, the demographic reality of Puerto Rico, where shrinking kin networks and large-scale outmigration have reduced the availability of family care, suggests that informal support alone may no longer be sufficient. In this context, improving the accessibility of formal or community-based services may be essential to supplement reduced family caregiving capacity and to strengthen practical sources of support for dementia caregivers.

The results should be interpreted with caution due to several limitations. First, while studies using the SSC model incorporate measures such as familism to capture culturally specific values and explain variation across ethnic groups, this study did not include such items or comparative ethnic groups. Future research should incorporate measures of cultural values to better capture potential within-group and between-group variation among Puerto Rican caregivers. Although we could not empirically test these cultural values with our data, they can help interpret the findings within the sociocultural context of dementia caregiving in Puerto Rico, where the majority of the population identifies as Hispanic. Second, while the LSNS-6 assesses network size and the number of available support resources, it may not capture the actual amount of support caregivers receive or their perceived satisfaction with that support (Haber et al., 2007). Future research may more effectively measure the construct of perceived social support by using instruments that capture caregivers’ experiences of received support, allowing for a more accurate understanding of the effects of social support.

Third, we were not able to examine differences by caregiver-care recipient relationship type due to the relatively small sample size and the fact that most caregivers in our sample were adult children. Prior evidence shows that spousal caregivers are generally more vulnerable to burden than adult children because they are viewed as the primary attachment figure, tend to assume a higher level of responsibility, are more likely to co-reside with the care recipient, and often have poorer physical health, all of which heighten the overall caregiving burden (Pinquart & Sörensen, 2011). Future research should therefore include the type of caregiver–care recipient relationship and, further, incorporate qualitative indicators that capture the characteristics of the relationship (e.g., marital satisfaction, emotional closeness). Including these variables would allow for a more nuanced understanding of how relationship characteristics shape caregiving appraisal and, in turn, influence mental health outcomes. Finally, since this study used cross-sectional data, it is difficult to determine the directionality or causality of the relationships among the variables.

Our study has several strengths. First, it addresses a significant gap in the literature by focusing on dementia caregivers in Puerto Rico, an underrepresented population in caregiving and mental health research. Second, it extends prior research that identified caregiver burden and BPSD as independent predictors of depressive symptoms by providing empirical evidence that perceptual disturbances/apathy, particularly symptoms related to appetite/eating, can magnify the association between caregiver burden and depressive symptoms. Third, it highlights the possibility that appetite/eating-related symptoms, a symptom often overlooked in previous BPSD studies, may be a key contributor to depressive vulnerability among Puerto Rican dementia caregivers.

In conclusion, we found that both caregiver burden and BPSD were strongly associated with depressive symptoms among dementia caregivers in Puerto Rico, even after adjusting for sociodemographic characteristics, caregiving hours, and perceived social support as covariates. We also found that one BPSD factor, perceptual disturbances/apathy, moderated this association, and further analyses suggested that symptoms related to appetite and eating primarily drove this result. Finally, we observed that perceived social support did not reduce the magnitude of caregiver burden on depressive symptoms, likely because social network availability did not translate into enacted support within the sociocultural and demographic context of Puerto Rico. Therefore, improving access to formal or community-based services may be critical to offset limited family caregiving capacity and provide more tangible support for dementia caregivers, particularly for those facing intensive demands related to BPSD.

## Supplementary Material

gbag014_Supplementary_Data

## Data Availability

The data utilized in the current study are available from Dr. Michael Crowe (e-mail: mgcrowe@uab.edu). De-identified data will be provided to individuals with prior approval or a “Non-Human Subjects Research” determination from their IRB. This study was not preregistered.
