# Correction to: Burden and well-being among dementia caregivers in Puerto Rico: the role of behavioral and psychological symptoms of dementia

**DOI:** 10.1093/geronb/gbag084

**Published:** 2026-05-19

Junyub Lim, Ross Andel, Frank Puga, María P Aranda, Maricruz Rivera-Hernandez, Ana Luisa Dávila-Roman, Michael Crowe. Burden and well-being among dementia caregivers in Puerto Rico: the role of behavioral and psychological symptoms of dementia *The Journals of Gerontology, Series B: Psychological Sciences and Social Sciences*, Volume 81, Issue 4, April 2026, gbag014 https://doi.org/10.1093/geronb/gbag014

In the originally published version of this article, an incorrect NIH R01 grant number was listed in the funding acknowledgment. The correct grant number is:

R01 AG064769

This error has been corrected.

