# Supplementary material for: Burden and well-being among dementia caregivers in Puerto Rico: the role of behavioral and psychological symptoms of dementia
Source: J Gerontol B Psychol Sci Soc Sci. 2026 Feb 5;81(4):gbag014. doi: 10.1093/geronb/gbag014 (PMC13017666; doi:10.1093/geronb/gbag014)
Supplement: Supplementary file 1 [file gbag014_supplementary_data.zip › JGPS suppl Lim, Andel, et al.docx]

***The Journals of Gerontology, Series B: Psychological Sciences and Social Sciences* Supplementary Material: Lim, Andel, Puga, Aranda, Rivera-Hernandez, Dávila-Roman, & Crowe. (2026). Burden and well-being among dementia caregivers in Puerto Rico: The role of behavioral and psychological symptoms of dementia.**

**Supplementary Figure 1:** *The conceptual framework of the relationship between caregiver burden and depressive symptoms*

**Supplementary Table 1:** *Correlational matrix for BPSD*

| **Variable** | **1.** | **2.** | **3.** | **4.** | **5.** | **6.** | **7.** | **8.** | **9.** | **10.** | **11.** | **12.** |
| --- | --- | --- | --- | --- | --- | --- | --- | --- | --- | --- | --- | --- |
| **1. Delusions** | -- |  |  |  |  |  |  |  |  |  |  |  |
| **2. Hallucinations** | .45*** | -- |  |  |  |  |  |  |  |  |  |  |
| **3. Agitation/Aggression** | .75*** | .38*** | -- |  |  |  |  |  |  |  |  |  |
| **4. Dysphoria/Depression** | .35** | .40*** | .26* | -- |  |  |  |  |  |  |  |  |
| **5. Anxiety** | .63*** | .39** | .31* | .39*** | -- |  |  |  |  |  |  |  |
| **6. Euphoria/Elation** | .33* | .07 | .22 | .19 | .25 | -- |  |  |  |  |  |  |
| **7. Apathy/Indifference** | .17 | .48*** | .24* | .31** | .22 | .10 | -- |  |  |  |  |  |
| **8. Disinhibition** | .70*** | .44*** | .67*** | .34** | .41*** | .27* | .30* | -- |  |  |  |  |
| **9. Irritability/Lability** | .54*** | .46*** | .58*** | .46*** | .56*** | .28* | .38*** | .60*** | -- |  |  |  |
| **10. Aberrant Motor** | .50*** | .21 | .34** | -.05 | .34** | .33** | .37*** | .44*** | .51*** | -- |  |  |
| **11. Nighttime Behavior** | .47*** | .49*** | .48*** | .23* | .51*** | .28* | .43*** | .45*** | .42*** | .37** | -- |  |
| **12. Appetite/Eating** | .15 | .18 | .20 | .49*** | .29* | .08 | .36*** | .24 | .15 | .06 | .29* | -- |

* *p* < .05, ** *p* < .01, *** *p* < .001.

**Supplementary Table 2:** *Standardized factor loadings of BPSD from an Exploratory Factor Analysis*

| **Variable** | **Factor 1:  Emotional Dysfunction/Behavioral Instability** | **Factor 2:  Perceptual Disturbances/Apathy** |
| --- | --- | --- |
| Delusions | .85 | .20 |
| Hallucinations | .40 | .50 |
| Agitation/Aggression | .71 | .22 |
| Dysphoria/Depression | .14 | .73 |
| Anxiety | .52 | .40 |
| Euphoria/Elation | .37 | .08 |
| Apathy/Indifference | .25 | .50 |
| Disinhibition | .73 | .30 |
| Irritability/Lability | .66 | .38 |
| Aberrant Motor | .63 | .02 |
| Nighttime Behavior | .52 | .40 |
| Appetite/Eating | .04 | .60 |
| Eigen values | 3.51 | 2.03 |
| Explained variance (%) | 29 | 17 |
| Cumulative variance (%) | 29 | 46 |
| Cronbach’s α | .80 | .71 |

*Note:* BPSD = behavioral and psychological symptoms of dementia.

**Supplementary Table 3:** *Correlational matrix for all study variables*

| **Variable** | **1.** | **2.** | **3.** | **4.** | **5.** | **6.** | **7.** | **8.** | **9.** | **10.** | **11.** |
| --- | --- | --- | --- | --- | --- | --- | --- | --- | --- | --- | --- |
| **1. Age** | -- | -- | -- | -- | -- | -- | -- | -- | -- | -- | -- |
| **2. Sex** | .03 | -- | -- | -- | -- | -- | -- | -- | -- | -- | -- |
| **3. Educational Attainment** | -.09 | .02 | -- | -- | -- | -- | -- | -- | -- | -- | -- |
| **4. Marital Status** | -.03 | .17 | -.05 | -- | -- | -- | -- | -- | -- | -- | -- |
| **5. Self-rated Health** | -.13 | -.09 | .30** | .11 | -- | -- | -- | -- | -- | -- | -- |
| **6. Perceived Social Support** | .02 | .11 | .14 | -.10 | -.15 | -- | -- | -- | -- | -- | -- |
| **7. Weekly caregiving hours** | .28** | -.15 | -.11 | -.25* | -.18 | -.02 | -- | -- | -- | -- | -- |
| **8. Depressive Symptoms** | .25* | .06 | .09 | .03 | -.25* | -.18 | .07 | -- | -- | -- | -- |
| **9. Caregiver Burden** | .11 | .07 | .12 | -.15 | -.23* | -.10 | .13 | .72*** | -- | -- | -- |
| **10. Factor 1** | .08 | .04 | .23* | .10 | -.13 | .00 | -.09 | .55*** | .50*** | -- | -- |
| **11. Factor 2** | .12 | .12 | .21* | .08 | -.11 | -.04 | -.12 | .57*** | .55*** | .84*** | -- |

*Notes:* Factor 1 =Emotional Dysfunction/Behavioral Instability; Factor 2 =Perceptual Disturbances/Apathy.

* *p* < .05, ** *p* < .01, *** *p* < .001.

**Supplementary Table 4:** Interaction effects between caregiver burden and each of the 12 BPSD in relation to caregiver depressive symptoms

|  | Model 1 | | | | Model 2 | | | | Model 3 | | | |
| --- | --- | --- | --- | --- | --- | --- | --- | --- | --- | --- | --- | --- |
|  | Est. | SE | β | *p* value | Est. | SE | β | *p* value | Est | SE | β | *p* value |
| Caregiver Burden * Delusions | 0.05 | 0.04 | 0.23 | .188 | 0.06 | 0.04 | 0.25 | .171 | 0.04 | 0.04 | 0.18 | .320 |
| Caregiver Burden * Hallucinations | 0.01 | 0.03 | 0.08 | .593 | 0.02 | 0.03 | 0.10 | .519 | 0.01 | 0.03 | 0.06 | .708 |
| Caregiver Burden * Agitation/Aggression | 0.03 | 0.02 | 0.18 | .209 | 0.03 | 0.02 | 0.19 | .192 | 0.03 | 0.02 | 0.16 | .248 |
| Caregiver Burden * Dysphoria/Depression | 0.05 | 0.03 | 0.25 | .096 | 0.05 | 0.03 | 0.26 | .089 | 0.06 | 0.03 | 0.29 | .055 |
| Caregiver Burden * Anxiety | -0.02 | 0.03 | -0.10 | .610 | -0.02 | 0.03 | -0.09 | .617 | -0.03 | 0.03 | -0.15 | .433 |
| Caregiver Burden * Euphoria/Elation | -0.02 | 0.04 | -0.05 | .720 | -0.02 | 0.04 | -0.05 | .693 | -0.04 | 0.05 | -0.13 | .353 |
| Caregiver Burden * Apathy/Indifference | 0.02 | 0.03 | 0.10 | .531 | 0.02 | 0.03 | 0.11 | .475 | 0.01 | 0.03 | 0.07 | .647 |
| Caregiver Burden * Disinhibition | 0.03 | 0.03 | 0.14 | .360 | 0.03 | 0.03 | 0.16 | .327 | 0.03 | 0.03 | 0.16 | .321 |
| Caregiver Burden * Irritability/Lability | 0.01 | 0.03 | 0.03 | .819 | 0.01 | 0.03 | 0.04 | .802 | 0.00 | 0.03 | 0.02 | .858 |
| Caregiver Burden * Aberrant Motor | 0.03 | 0.03 | 0.14 | .324 | 0.03 | 0.03 | 0.14 | .336 | 0.02 | 0.04 | 0.07 | .636 |
| Caregiver Burden * Nighttime behavior | 0.02 | 0.03 | 0.10 | .527 | 0.02 | 0.03 | 0.13 | .429 | 0.01 | 0.03 | 0.08 | .609 |
| Caregiver Burden * Appetite/ Eating | 0.08 | 0.03 | 0.43 | .007 | 0.08 | 0.03 | 0.42 | .007 | 0.07 | 0.03 | 0.37 | .020 |

*Notes:* Est. = Estimate (unstandardized regression coefficient); SE = standard error; β = standardized regression coefficient. Model 1 was adjusted for age, sex, education, marital status, self-rated health; Model 2 was also adjusted for hours of caregiving per week; Model 3 was also adjusted for perceived social support.
